# The interactive relationship of dietary choline and betaine with physical activity on circulating creatine kinase (CK), metabolic and glycemic markers, and anthropometric characteristics in physically active young individuals

**DOI:** 10.1186/s12902-023-01413-3

**Published:** 2023-07-25

**Authors:** Ensiye Soleimani, Abnoos Mokhtari Ardekani, Ehsan Fayyazishishavan, Mahdieh Abbasalizad Farhangi

**Affiliations:** 1 Department of Community Nutrition, Faculty of Nutrition, Tabriz University of Medical Sciences, Tabriz, Iran; 2 Endocrinology and Metabolism Research Center, Institute of Basic and Clinical Physiology Science, & Physiology Research Center, Kerman University of Medical Sciences, Kerman, Iran; 3 Department of Biostatistics and Data Science, School of Public Health, The University of Texas Health Science Center at Houston (UTHealth), Houston, TX77030 USA; 4 Tabriz Health Services Management Research Center, Tabriz University of Medical Sciences, Tabriz, Iran

**Keywords:** Choline, Betaine, Physical activity, Insulin resistance, Lipid profile

## Abstract

**Background:**

There is conflicting evidence on the relationship between dietary choline and betaine with metabolic markers and anthropometric characteristics. The aim of this study is to investigate the relationship between the interaction effects of dietary choline and betaine and physical activity (PA) on circulating creatine kinase (CK), metabolic and glycemic markers, and anthropometric characteristics in active youth.

**Methods:**

In this cross-sectional study, data were collected from 120 to 18 to 35-year-old people. The food frequency questionnaire was used to assess dietary data; United States Department of Agriculture website was used to calculate choline and betaine in foods. CK, fasting blood sugar (FBS) and lipid profile markers were measured with ELISA kits. Low-density lipoprotein, and insulin sensitivity markers were calculated. Sociodemographic status, physical activity, and anthropometric characteristics were assessed based on a valid and reliable method. Analysis of co-variance (ANCOVA) tests adjusted for sex, PA, age, energy, and body mass index were used.

**Results:**

Increasing dietary betaine and total choline and betaine was positively related to weight, waist-to-hip ratio, fat-free mass and bone mass (*P* < 0.05). Increasing dietary betaine lowered total cholesterol (*P* = 0.032) and increased high density lipoprotein (HDL) (*P* = 0.049). The interaction effect of dietary choline and physical activity improved insulin resistance (*P* < 0.05). As well as dietary betaine interacted with physical activity increased HDL (*P* = 0.049). In addition, dietary total choline and betaine interacted with physical activity decreased FBS (*P* = 0.047).

**Conclusions:**

In general, increasing dietary choline and betaine along with moderate and high physical activity improved insulin resistance, increased HDL, and lowered FBS in the higher tertiles of dietary choline and betaine.

**Supplementary Information:**

The online version contains supplementary material available at 10.1186/s12902-023-01413-3.

## Background

The evidence and results of medical research show that having regular physical activity as one of several components of a healthy lifestyle is the most effective and accepted way to prevent the rise of noncommunicable diseases [1, 2]. For adults to earn health benefits, 75 min/week of intensive activity or 150 min/week of normal-intensity aerobic physical activity is suggested. There is evidence that recommended amounts of activity can reduce the hazard of some kinds of cancer, obesity, high blood pressure, osteoporosis, cardiovascular disease, diabetes, anxiety, stress, and depression [3–5]. Because of the importance of proper dietary choices in people who engage in regular physical activity, some nutrients have received special attention [4, 6–8]. Choline and betaine are essential nutrients obtained from the diet or de novo synthesis, and they are rich in different kinds of foods. Foods high in choline include liver, eggs, pork, seafood, milk, and beef, while seeds, grains, spinach, and beets are high in betaine [9–12]. As a methyl donor and precursor for phospholipids, lipoproteins, and acetylcholine, choline plays a critical role in cell membrane signaling, lipid transport, and neurotransmitter synthesis [9, 13, 14]. The derivative of choline, betaine, can serve as a methyl donor in many ways, including methylating homocysteine [15]. As part of a neurotransmitter, choline is effective in transmitting messages to skeletal muscles and thus plays a role in physical activity [16]. Inadequate dietary choline leads to decreased choline levels, followed by several changes in myoblasts, including muscle wasting, and finally increased serum creatine kinase (CK). There may be a relationship between insufficient of dietary choline and increase serum CK [17–19].

Studies have demonstrated that reducing weight, increasing activity, and changing eating habits or dietary components can increase glucose tolerance and modify lipid profiles [4, 20–23]. There has been conflicting evidence regarding the effects of dietary choline and betaine on glucose tolerance. Choline deficiency may modify glucose tolerance in some studies, but increasing dietary choline and betaine may also increase insulin sensitivity [6, 24–26]. The liver needs choline to transport triglycerides and very low-density lipoproteins (VLDL) into the blood. Although some evidence has been established regarding choline’s role in lipid metabolism, few studies have examined its association with human lipid profiles [27, 28]. To date, the relationship between the interaction effects of dietary choline and betaine with physical activity on changes in blood sugar and lipid profiles has not been investigated in any study.

The identification of anthropometric measurements is a useful method for studying the nutritional status of adults. Regular physical activity is related to better anthropometric measurements in youth [29, 30]. Among them are: weight loss, fat reduction, decreased waist-to-hip ratio, and waist circumference [31]. The relationship between higher dietary choline and betaine with favorable anthropometric characteristics, such as lower waist circumference (WC), lower body fat %, and weight loss, has been investigated in different studies [32–35]. However, additional investigation is required to understand the interaction effects of dietary choline and betaine and physical activity on these parameters. The aim of this study is to investigate the relationship between the interacted effects of dietary choline and betaine and physical activity (PA) on circulating CK, metabolic and glycemic markers, and anthropometric traits in active youth.

## Methods

### Participant population

**Fig. 1:**
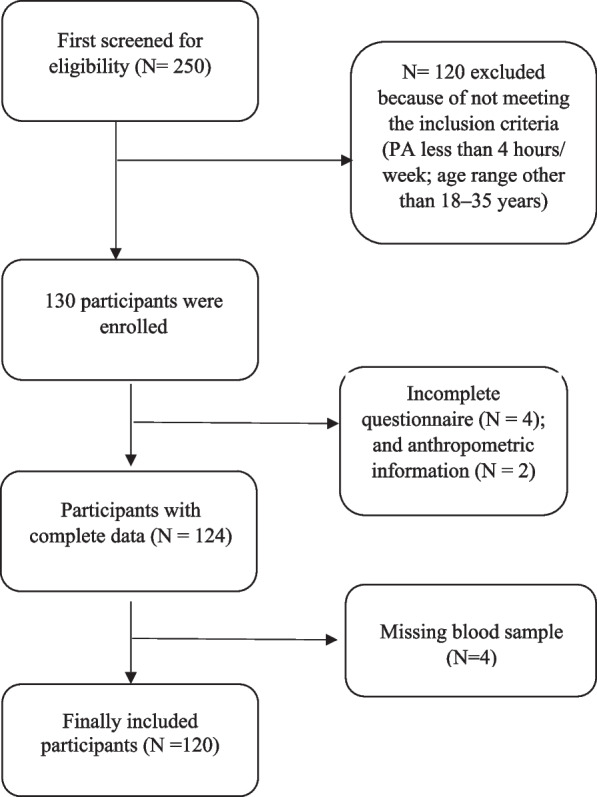
Study flowchart

A total of 120 young people from Tabriz participated in this cross-sectional study (Fig. 1). HOMA-IR (homeostatic model assessment for insulin resistance) was used to calculate the sample size [6], Z = 1.96, E = 8% mean, SD = 1.49 using the following formula: *n* = (z^2^×sd^2^)/e^2^ the total number of samples reached 120, considering 10% missing. Inclusion criteria are as follows: preparing to participate in the project, moderate physical activity for at least 4 h per week; age range 18–35 years; blood sampling was done 24 h after the last exercise to prevent acute changes in plasma volume caused by exercise that affect serum creatine kinase levels [18]; and having the physical ability to complete the measurement process and fill out the questionnaires. Exclusion criteria also included chronic conditions affecting food intake, including digestive problems and cancer, anorexia, alcohol and drug use, and the use of drugs and dietary supplements containing choline and betaine.

### Dietary intake and choline estimate

Food data were collected through a food frequency questionnaire whose validity and reliability was proven by Nik Niaz et al.; this questionnaire was adjusted for the Iranian population [36]. Every participant is asked to report accurately and honestly how much and how often they consume each food item on a yearly, monthly, weekly, or daily basis. Based on household standards, participants’ dietary intakes were converted into grams. An Iranian diet was adapted using NUTRITIONIST 4 software (version 7.0; N Squared Computing, Salem, OR), and calorie and nutrient intakes were calculated. Each gram of each food was multiplied by the amount of choline and betaine in each gram based on tables in the USDA database [37–39]. To calculate the total dietary choline, different forms of choline (phosphatidylcholine, phosphocholine, free choline, and glycerophosphocholine) were considered. For total choline and betaine, we summed the columns of total dietary choline and betaine. The table provided by USDA includes different types of foods with different preparation methods and indicates the choline content per 100 g of the food. We calculated this amount for each gram of a food that is closest to Iranian food culture in terms of cooking and storage methods and used it in the next steps. For example, in beef and beef products, there are about 37 different types of beef with different fat percentages and preparation methods. In Iranian culture, this type of meat is mostly used as a stew, so for the further calculations, the moderately fat-cooked type was used, which was selected based on the closest similarity. For eggs, the USDA food table lists 5 items, two of which are fried and two of which are boiled, and in this case, we calculated the average of these two and used it for the next steps [37, 38, 40–42].

### Sociodemographic, anthropometric, and physical activity measurements

Sociodemographic information such as marital status, age, gender, and education level was gathered by questionnaire. Anthropometric features such as participant body mass index (BMI), weight, height, waist-to-hip ratio (WHR), hip circumference (HC), WC, mid-arm circumference (MAC), thigh circumference, and leg circumference were assessed. A Seca 753E electronic scale was used to precisely measure the subject’s weight (down to 0.1 kg) over a minimal range. The standing height of the participants was measured in a stationary position and without shoes (accurate to 0.1 cm), and then the BMI was computed (kg/m2) based on the formula. While the participants were lightly dressed, WC was measured as the distance between the smallest area below the ribs and above the iliac spine. Maximum HC was measured as hip circumference. The middle point between the elbow and shoulder distance was measured as the MAC. Approximately 10 cm below the groin was measured as the largest circumference of the thigh. The largest calf circumference was measured as calf circumference. All measurements will be measured with the help of an inflexible tape measure without any pressure on the meter with an accuracy of 0.1 cm. Body composition measurements were recorded while the participants were fasting for 12 h. While wearing light clothing, subjects were weighed after emptying their bladders using a bioelectrical impedance analysis (BIA) machine (Tanita, BC-418 MA, Tokyo, Japan). AIPAC or the short 7-item international physical activity questionnaire (IPAQ) was used to determine physical activity levels worldwide [30]. The short form of this questionnaire consists of 7 simple questions (validity and reliability having already been measured).

### Biochemical measurements

First, 5 cc of venous blood samples from the participants were drawn by a laboratory technician while fasting for 8 to12 hours and then centrifuged for 10 min to separate serum and plasma. Creatine kinase (CK), fasting insulin, total cholesterol (TC), triglycerides (TG), and high-density lipoprotein cholesterol (HDL-C) were tested by spectrophotometry and ELISA (enzyme-linked immunosorbent assay). Low-density lipoprotein (LDL-C) was also calculated using the Friedwald Eq. [43]. The homeostatic model of insulin resistance (HOMA-IR) was assessed using the formula fasting insulin (µIU/mL), fasting blood glucose (mmol/L)/22.5, and quantitative insulin sensitivity index (QUICKI) as 1/[log fasting insulin (mU/L) + log (fasting plasma glucose (mmol/L)×18.0182)] [44–46].

### Analyzing data statistically

Statistical analysis of the collected data was performed using SPSS software (version 21.0; SPSS Inc, Chicago, IL). A *P* value of less than 0.05 was considered significant. Normality was tested with the Shapiro-Wilk test (P > 0.05), and Levene’s test (P > 0.05) for equality of error variance was used to test for equality of variance. The mean standard deviation (SD) was used to indicate quantitative data, and numbers and percentages (%) were used for qualitative data. Differences between discrete and continuous variables in the different tertiles of dietary choline, betaine, and total choline and betaine were compared using the chi-square test and one-way analysis of variance (ANOVA), respectively. Bonferroni’s post hoc multiple comparison analysis revealed a significant mean difference between groups. Analysis of covariance (ANCOVA), general linear model (GLM), and univariate analysis were used to compare the biochemical and anthropometric variables after adjustment for confounding factors such as age and sex, BMI, PA, and energy intake. Additionally, the same method was used to investigate the interaction effect of dietary choline and betaine and physical activity and biochemical characteristics.

## Results

**Fig. 2:**
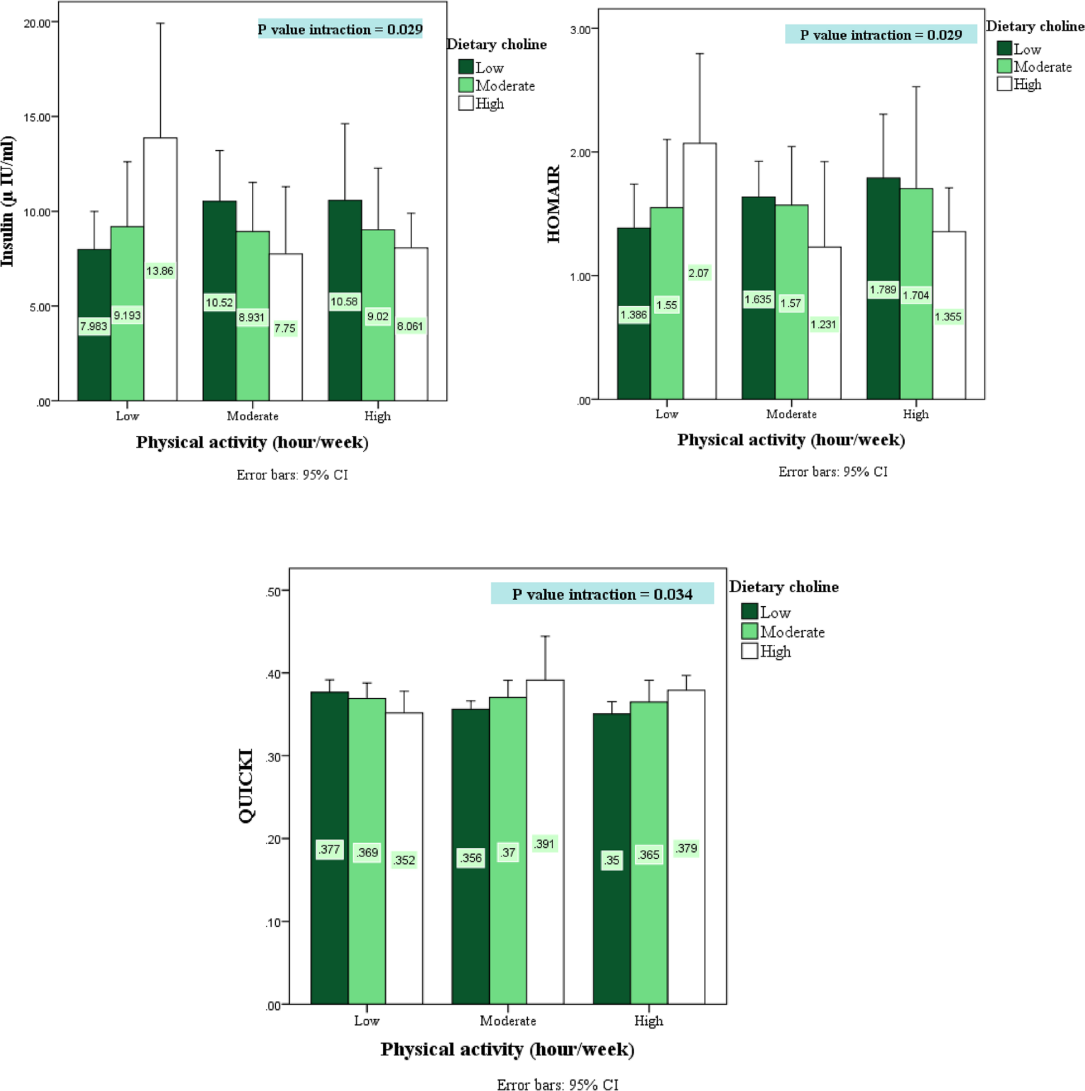
The interaction between dietary choline and physical activity on biochemical markers. HOMA-IR; Homeostatic model assessment for insulin resistance; QUICKI; quantitative insulin-sensitivity check index. *P* values of interaction are obtained from ANCOVA model after adjustment for the confounding effects of age, sex, BMI and calorie intake. *P Significant at *P* < .05; 95th confidence intervals of the difference in parentheses

**Fig. 3:**
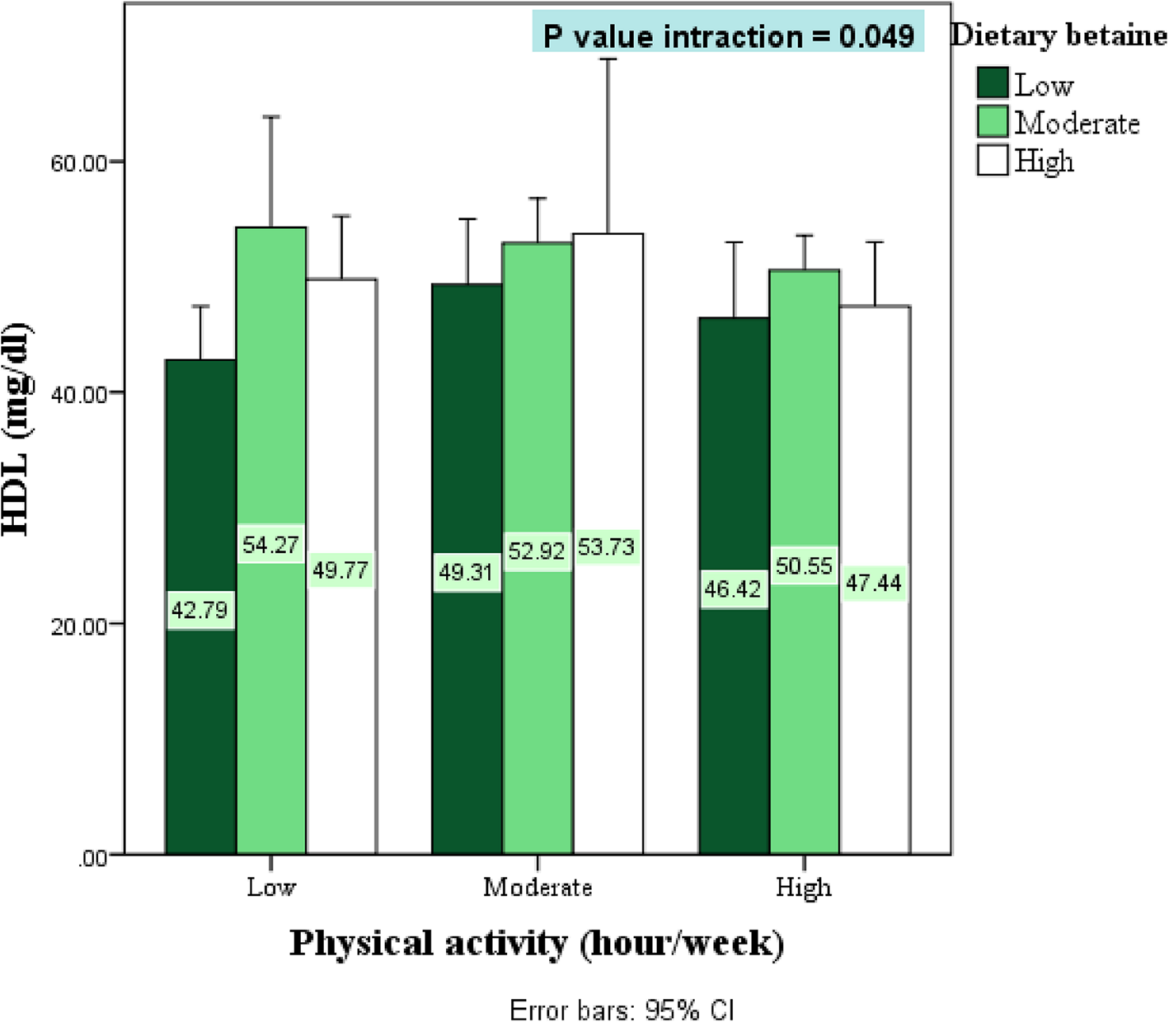
The interaction between dietary betaine and physical activity on biochemical markers. HDL; high density lipoprotein. *P* values of interaction are obtained from ANCOVA model after adjustment for the confounding effects of age, sex, BMI and calorie intake*P Significant at *P* < .05; 95th confidence intervals of the difference in parentheses

**Fig. 4:**
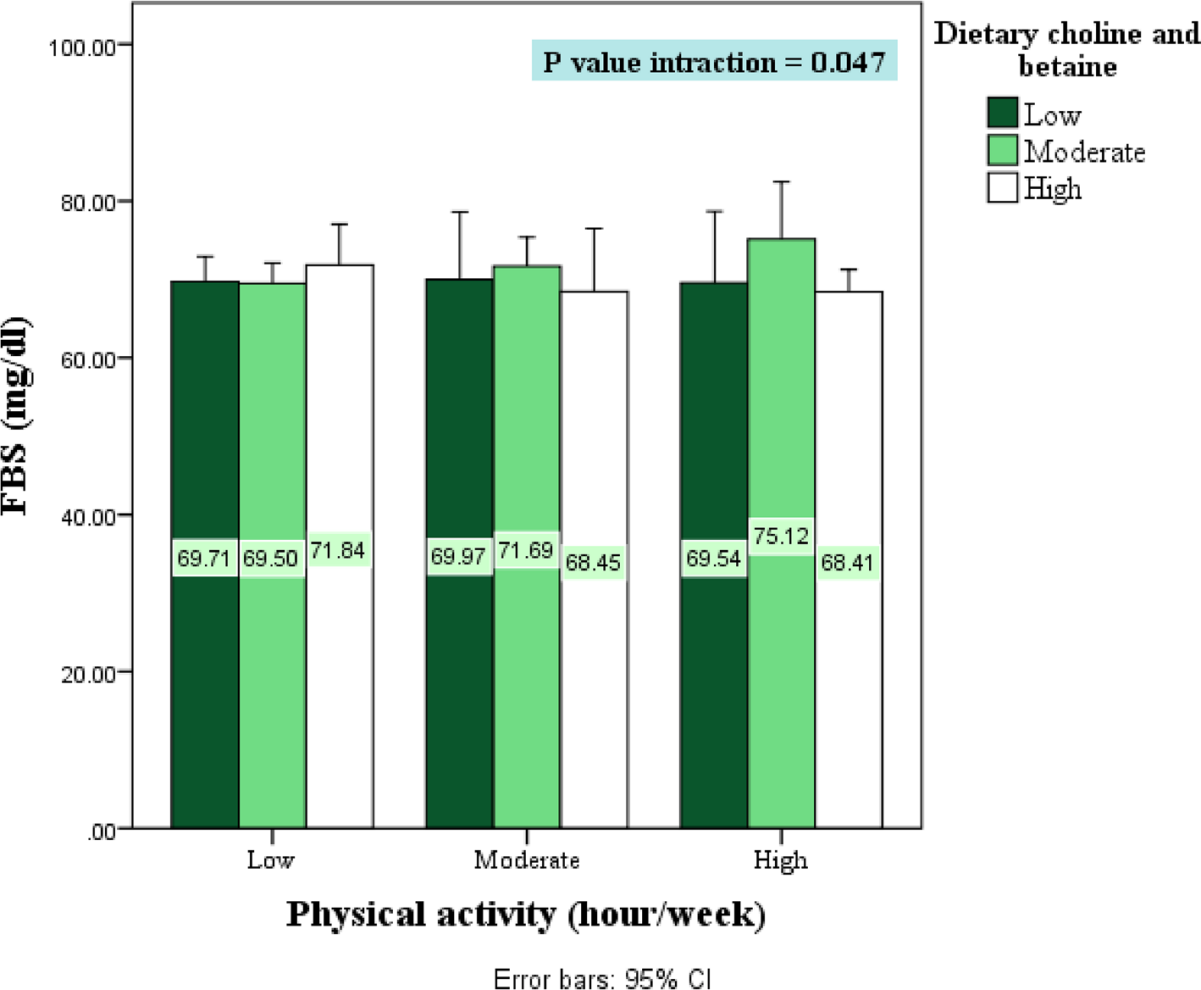
The interaction effect of dietary choline + betaine and physical activity on biochemical markers. FBS; fasting blood sugar. *P* values of interaction are obtained from ANCOVA model after adjustment for the confounding effects of age, sex, BMI and calorie intake. *P Significant at *P* < .05; 95th confidence intervals of the difference in parentheses

A total of 250 individuals were screened for eligibility and 130 were found to be eligible for participation in the current study. However, 10 individuals were excluded due to incomplete biochemical, anthropometric or dietary data. Therefore, 120 individuals were completed the study (Fig. 1). General characteristics and anthropometric measurements of study participants across different tertiles of dietary choline, betaine, and total choline and betaine are shown in Table 1. After controlling for potentially confounding variables (age, sex, BMI, energy intake, PA), an increase in dietary betaine was significantly related to an increase in weight (*P* = 0.045), height (*P* = 0.001), MAC (*P* = 0.038) and WHR (*P* = 0.044). According to Bonferroni’s post hoc test, the average WHR difference was between T3 and T2 dietary betaine (*P* = 0.019). Additionally, there was a significant direct relationship between total dietary choline and betaine and weight (*P* = 0.038), WC (*P* = 0.005), and WHR (*P* = 0.011). According to Bonferroni’s post hoc test, the average weight (*P* = 0.021), WHR (*P* = 0.034), and HC (*P* = 0.005) differences were between T3 and T2 dietary total choline and betaine and about MAC (*P* = 0.007) the difference was between T3 and T1 (Table 1). Additionally, after considering the confounding variables, the increase in dietary betaine was related to the increase in FFM (*P* = 0.002), MM (*P* = 0.002), and BM (*P* = 0.001). Additionally, the increase in dietary choline and betaine was directly related to the increase in FFM (*P* = 0.047) and BM (*P* = 0.041). The average difference in FFM (*P* = 0.049), MM (*P* = 0.033) and BM (*P* = 0.044) between T3 and T1 was determined by Bonferroni’s post hoc test (Table 2). Additionally, after considering the confounding variables, the increase in dietary choline was related to the increase in the intake of calcium (*P* = 0.015), vitamin B8 (*P* = 0.043) and vitamin D (*P* = 0.037). Additionally, the increase in dietary betaine was related to protein (*P* = 0.007) and fat (*P* < 0.001), vitamin B1 (*P* < 0.001), vitamin B3 (*P* < 0.001), polyunsaturated fatty acid (PUFA) (*P* = 0.044), monounsaturated fatty acid (MUFA) (*P* < 0.001), and saturated fatty acid (SAFA) (*P* = 0.007) intake. There was also a direct relationship between dietary choline and betaine and vitamin B3. (*P* = 0.044) (Table 3). After considering the confounding variables, the increase in dietary betaine was related to the decrease in total cholesterol (*P* = 0.032) and increase in HDL (*P* = 0.049). According to Bonferroni’s post hoc test, the average HDL in the T2 dietary betaine had significant differences compared to T1 (*P* = 0.009) and TC in the T3 dietary betaine had a significant difference compared to T2 (*P* = 0.051). After considering all the confounding variables, the increase in dietary choline was associated with the increase in serum creatine kinase (*P* = 0.063), although this relationship was not significant (Table 4). The interaction between dietary choline and physical activity on biochemical markers is presented (Fig. 2). Subjects were divided into three categories based on the duration of their physical activity per week: less than 5 h (low), between 5 and 8 h (moderate), and more than 8 h (high). At moderate and high physical activity levels, increased dietary choline decreased insulin (*P* = 0.029) and HOMA-IR (*P* = 0.029) and increased QUICKI (*P* = 0.034). The interaction between dietary betaine and physical activity on biochemical markers is shown (Fig. 3). At all levels of physical activity, HDL levels were significantly higher in the moderate betaine intake group than in the lowest intake group. Even in the moderate physical activity group, the HDL trend was completely increasing with increasing dietary betaine (*P* = 0.049). The interaction between dietary choline and betaine and physical activity on biochemical markers is shown (Fig. 4). In the group that had the most physical activity, the highest level of dietary choline and betaine decreased FBS levels (*P* = 0.047). For the other biochemical variables, the interaction between dietary choline, betaine or combination of both was non-significant (Sup. Figures 1, 2, 3).

**Table 1:** General characteristics and anthropometric measurements of study participants across different tertiels of dietary choline, betaine and total choline and betaine intake

Variables	Total choline					Total betaine					Total choline and betaine				
	T1 (*n* = 40)	T2 (*n* = 40)	T3 (*n* = 40)	*P	**P	T1 (*n* = 40)	T2 (*n* = 40)	T3 (*n* = 40)	*P	**P	T1 (*n* = 40)	T2 (*n* = 40)	T3 (*n* = 40)	*P	**P
**Age (year)**	25.13(4.02)	22.97(4.07)	22.48(3.48)	0.007	0.104	24.39(4.32)	23.22(4.03)	22.92(3.58)	0.233	0.743	25.10(4.46)	22.67(3.49)	22.75(3.60)	0.008	0.067
**Gender (male %)**	15(39.5)	20(50.0)	28(68.3)	0.010	-	11(28.9)	19(47.5)	33(80.5)	< 0.001	-	12(31.6)	22(56.4)	29(70.7)	0.001	-
**Marital status (single %)**	34(89.5)	36(90.0)	38(92.7)	0.621	-	34(89.5)	37(92.5)	37(90.2)	0.915	-	34(89.5)	36(92.3)	37(90.2)	0.914	-
**Education (university graduate %)**	21(55.3)	6(15.0)	7(17.1)	< 0.001	-	14(36.8)	12(30.0)	8(19.5)	0.089	-	19(50.0)	8(20.5)	6(14.6)	0.001	-
**Occupation status (student %)**	32(84.2)	35(87.5)	35(85.4)	0.891	-	31(81.6)	36(90.0)	35(85.4)	0.647	-	31(81.6)	34(87.2)	36(87.8)	0.438	-
**Weight (kg)**	68.43(15.11)	68.01(13.45)	74.68(13.91)	0.064	0.622	65.38(14.91)	69.31(12.63)	76.24(13.71)	0.002	**0.045**^b^	66.30(14.62)	67.08(11.34)	77.85(14.25)	< 0.001	**0.038**^b^
**Height (cm)**	169.80(9.35)	169.26(10.00)	175.93(8.38)	0.002	0.347	166.96(8.81)	170.41(8.22)	177.45(9.04)	< 0.001	**0.001**^b^	167.94(8.76)	170.53(9.35)	176.62(9.00)	< 0.001	0.183
**BMI (kg/m2)**	23.42(3.77)	23.45(3.42)	23.82(3.83)	0.862	0.967	23.11(3.74)	23.62(3.69)	23.96(3.57)	0.586	0.670	23.20(3.65)	22.90(3.37)	24.61(3.78)	0.083	0.161
**MAC (cm)**	28.23(3.96)	28.33(3.25)	29.54(3.67)	0.202	0.883	27.36(3.68)	28.65(3.41)	30.04(3.45)	0.004	**0.038**^a^	27.78(3.81)	28.03(3.40)	30.32(3.27)	0.002	0.110
**WC (cm)**	77.76(9.83)	76.33(8.48)	80.28(8.18)	0.131	0.208	75.42(9.24)	79.20(9.21)	79.65(7.89)	0.070	0.115	76.73(9.42)	75.68(7.89)	82.00(8.27)	0.003	**0.005**^b^
**HC (cm)**	100.39(7.12)	99.51(6.82)	100.54(8.04)	0.794	0.817	99.46(7.02)	99.62(7.50)	101.30(7.41)	0.461	0.429	99.89(6.97)	98.40(6.70)	102.15(7.87)	0.068	0.149
**THC (cm)**	57.27(5.09)	56.43(5.80)	56.70(6.36)	0.809	0.480	57.46(5.40)	56.45(6.04)	56.52(5.86)	0.694	0.853	57.24(5.14)	55.73(5.80)	57.42(6.25)	0.359	0.162
**CC (cm)**	36.78(3.56)	36.75(3.75)	37.28(3.40)	0.760	0.810	36.53(3.69)	36.81(3.68)	37.45(3.31)	0.505	0.525	36.56(3.42)	36.55(3.66)	37.71(3.52)	0.284	0.333
**WHR (cm)**	0.77(0.06)	0.76(0.07)	0.79(0.04)	0.057	0.114	0.75(0.05)	0.79(0.06)	0.78(0.05)	0.020	**0.044**^b^	0.76(0.06)	0.77(0.06)	0.80(0.04)	0.012	**0.011**^b^
**PA (met-hour/week)**	5.52 (2.03)	7.82 (6.29)	11.22 (8.48)	< 0.001	0.703	6.13 (3.56)	7.99 (6.30)	10.48 (8.41)	0.013	0.874	5.65 (2.09)	7.94 (6.70)	11.11 (8.34)	0.001	0.839

*BMI *Body mass index, *MAC *Mid-arm circumference, *WC *Waist circumference, *HC *Hip circumference, *THC *Thigh circumference, *CC *Calf circumference, *WHR *Waist-to-hip ratio, *PA *Physical activity. Data are presented as mean ± SD or percent; *Obtained from the one-way analysis of variance (ANOVA) or Chi-squared tests, where appropriate; P Significant at *P* < 0.05; 95th confidence intervals of the difference in parentheses **Obtained from ANCOVA model after adjustment for the confounding effects of age, sex, BMI and physical activity, calorie intake; P Significant at *P* < 0.05; 95th confidence intervals of the difference in parentheses

^a^post hoc Tukey signature difference between 1st tertile and 3rd tertile

^b^post hoc Tukey signature difference between 2nd tertile and 3rd tertile

**Table 2:** Body composition parameters of study participants across different tertiels of dietary choline, betaine and total choline and betaine intake

Variables	Total choline					Total betaine					Total choline and betaine				
	T1 (*n* = 40)	T2 (*n* = 40)	T3 (*n* = 40)	*P	**P	T1 (*n* = 40)	T2 (*n* = 40)	T3 (*n* = 40)	*P	**P	T1 (*n* = 40)	T2 (*n* = 40)	T3 (*n* = 40)	*P	**P
**FM (kg)**	16.51 (7.84)	14.60 (6.25)	14.09 (7.48)	0.289	0.834	16.13 (7.85)	15.28 (6.52)	13.78 (7.26)	0.334	0.857	16.67 (7.84)	13.36 (5.21)	15.18 (8.10)	0.119	0.092
**FFM (kg)**	51.62 (10.67)	52.62 (11.12)	59.77 (11.11)	0.002	0.665	49.04 (10.43)	53.26 (9.68)	61.66 (10.71)	< 0.001	**0.002**^a^	49.79 (10.05)	53.14 (9.93)	61.04 (11.55)	< 0.001	**0.047**^a^
**FFM (%)**	76.28 (7.29)	78.37 (7.72)	81.30 (8.51)	0.018	0.901	75.79 (7.40)	78.02 (7.46)	82.13 (8.17)	0.001	0.116	75.49 (7.30)	80.14 (7.14)	80.53 (8.82)	0.007	0.160
**MM (kg)**	49.02 (10.17)	49.97 (10.60)	56.73 (10.59)	0.002	0.685	46.56 (9.95)	50.58 (9.22)	58.52 (10.23)	< 0.001	**0.002**^a^	47.28 (9.58)	50.46 (9.47)	57.94 (11.02)	< 0.001	0.064
**BM (kg)**	2.59 (0.49)	2.65 (0.51)	2.98 (0.51)	0.002	0.696	2.47 (0.48)	2.68 (0.45)	3.07 (0.49)	< 0.001	**0.001**^a^	2.51 (0.47)	2.67 (0.46)	3.04 (0.53)	< 0.001	**0.041**^a^
**SMM (kg)**	29.30 (6.46)	30.32 (7.16)	40.21 (32.27)	0.023	0.110	27.84 (6.47)	35.82 (32.76)	36.14 (6.99)	0.107	0.318	28.16 (6.11)	35.97 (32.78)	35.67 (7.56)	0.139	0.341
**FM (%)**	23.71 (7.29)	21.62 (7.71)	18.67 (8.53)	0.017	0.902	24.21 (7.39)	21.97 (7.45)	17.84 (8.20)	0.001	0.114	24.50 (7.29)	20.09 (7.19)	19.44 (8.85)	0.008	0.163
**MA (year)**	23.70 (12.46)	21.29 (9.26)	20.40 (9.41)	0.349	0.850	22.47 (12.21)	22.39 (10.03)	20.50 (9.10)	0.638	0.857	23.87 (12.45)	19.80 (8.71)	21.75 (9.81)	0.219	0.155

*FM *Fat mass, *FFM *Fat free mass, *MM *Muscle mass, *BM *Bone mass, *SMM *Skeletal muscle mass, *FP *Fat percent, *SMM *Skeletal muscle mass, *MA *Metabolic age; **P values are obtained from ANCOVA model after adjustment for the confounding effects of age, sex, BMI and physical activity, calorie intake *P Significant at *P* < 0.05; 95th confidence intervals of the difference in parentheses

^a^post hoc Tukey signature difference between 1st tertile and 3rd tertile

**Table 3:** Energy adjusted dietary intakes of study participants across different tertiels of dietary choline, betaine and total choline and betaine intake

Variables	Total choline					Total betaine					Total choline and betaine				
	T1 (*n* = 40)	T2 (*n* = 40)	T3 (*n* = 40)	*P	**P	T1 (*n* = 40)	T2 (*n* = 40)	T3 (*n* = 40)	*P	**P	T1 (*n* = 40)	T2 (*n* = 40)	T3 (*n* = 40)	*P	**P
**Energy (kcal)**	1659.85 (383.49)	2329.22 (535.13)	3809.73 (1279.29)	< 0.001	**< 0.00**^a^	1826.25 (704.35)	2515.91 (978.81)	3473.38 (1309.25)	< 0.001	**< 19460.001**^a^	1643.90 (389.20)	2384.81 (642.68)	3764.81 (1275.96)	< 0.001	**< 0.001**^a^
**Choline (mg/d)**	180.49 (33.38)	273.29 (30.94)	535.14 (318.71)	< 0.001	**< 0.001**^a^	220.62 (75.09)	302.18 (117.06)	469.75 (346.97)	< 0.001	**0.002**^a^	187.61 (38.56)	281.87 (61.01)	520.95 (327.35)	< 0.001	**< 0.001**^a^
**Betaine (mg/d)**	87.70 (40.83)	145.87 (85.23)	231.64 (149.73)	< 0.001	**< 0.001**^a^	65.98 (15.43)	120.49 (18.88)	276.52 (130.50)	< 0.001	**< 0.001**^a^	72.49 (23.24)	131.37 (42.18)	261.28 (143.21)	< 0.001	**< 0.001**^a^
**Protein (g/day)**	53.89 (11.59)	76.91 (13.71)	146.75 (64.34)	< 0.001	0.062	59.57 (19.71)	82.96 (26.92)	135.16 (71.18)	< 0.001	**0.007**^b^	53.34 (11.42)	78.99 (15.33)	145.21 (65.49)	< 0.001	0.086
**Fat (g/day)**	55.67 (16.97)	75.00 (21.50)	136.86 (73.31)	< 0.001	0.376	65.19 (27.96)	90.46 (49.41)	112.12 (73.87)	0.001	**< 0.001**^a,b^	56.93 (17.08)	78.17 (27.53)	131.74 (75.68)	< 0.001	0.196
**CHO (g/day)**	248.33 (71.34)	349.82 (97.69)	520.19 (178.15)	< 0.001	0.382	262.94 (105.91)	359.13 (141.34)	496.63 (160.16)	< 0.001	0.051	242.56 (67.73)	354.76 (108.08)	521.06 (169.85)	< 0.001	0.468
**Total Fiber (g/day)**	11.96 (3.56)	15.45 (5.10)	25.09 (13.11)	< 0.001	0.487	1318 (6.30)	17.35 (10.26)	22.01 (10.99)	< 0.001	0.125	11.97 (3.57)	15.87 (6.26)	24.65 (12.98)	< 0.001	0.355
**SFA (g/day)**	15.97 (4.63)	23.18 (6.93)	44.78 (30.22)	< 0.001	0.479	19.77 (8.89)	27.64 (14.55)	36.62 (31.72)	0.002	**0.007**	16.40 (5.11)	24.88 (8.66)	42.57 (31.29)	< 0.001	0.981
**MUFA (g/day)**	17.26 (6.29)	23.50 (8.16)	44.91 (26.36)	< 0.001	0.210	20.54 (10.09)	28.84 (16.37)	36.37 (27.09)	0.002	**< 0.001**^a,b^	17.82 (6.33)	24.43 (10.27)	43.21 (27.07)	< 0.001	0.070
**PUFA(g/day)**	14.75 (6.56)	17.73 (8.08)	27.33 (19.88)	< 0.001	0.146	16.29 (8.98)	21.96 (16.78)	21.60 (14.50)	0.125	**0.044**^a,b^	14.90 (6.53)	18.11 (9.44)	26.43 (19.64)	< 0.001	0.141
**Cholesterol (mg/day)**	107.77 (34.99)	150.32 (42.56)	371.78 (308.38)	< 0.001	0.410	127.54 (54.82)	175.54 (75.54)	327.28	< 0.001	0.472	106.98 (35.13)	161.29 (48.45)	360.32 (315.26)	< 0.001	0.508
**Sodium (mg/day)**	2164.46 (1159.97)	2829.14 (1233.60)	4116.87 (2422.48)	< 0.001	0.639	2358.54 (1135.83)	3132.64 (1647.18)	3624.03 (2430.84)	0.009	0.705	2267.61 (1168.74)	2732.41 (1268.54)	4080.90 (2444.37)	< 0.001	0.722
**Iron (mg/day)**	17.07 (9.14)	19.85 (5.29)	34.11 (20.00)	< 0.001	0.105	17.92 (8.42)	23.20 (19.05)	29.94 (13.16)	0.001	0.425	16.55 (9.03)	20.25 (5.60)	34.22 (19.82)	< 0.001	0.119
**Magnesium (mg/day)**	222.08 (40.13)	292.55 (61.42)	463.34 (211.35)	< 0.001	0.756	255.97 (74.08)	322.77 (136.72)	400.05 (216.14)	< 0.001	0.134	231.12 (45.49)	298.01 (78.24)	448.19 (219.58)	< 0.001	0.724
**Zinc (mg/day)**	6.13 (1.63)	8.28 (1.27)	15.81 (7.40))	< 0.001	0.065	7.33 (2.76)	9.71 (4.70)	13.21 (8.01)	< 0.001	0.262	6.28 (1.70)	8.70 (2.15)	15.18 (7.82)	< 0.001	0.546
**Phosphorus (mg/day)**	840.58 (213.10)	1161.90 (249.39)	2065.52 (926.56)	< 0.001	0.109	1017.93 (421.36)	1330.39 (620.42)	1724.01 (986.64)	< 0.001	0.165	867.00 (239.23)	1218.31 (348.64)	1968.70 (981.20)	< 0.001	0.924
**Calcium (mg/day)**	762.55 (221.64)	1112.60 (318.26)	1690.15 (767.92)	< 0.001	**0.015**^a^	928.56 (425.27)	1145.00 (491.60)	1495.79 (774.50)	< 0.001	0.916	797.70 (268.28)	1123.80 (419.93)	1639.44 (759.05)	< 0.001	0.132
**Potassium (mg/day)**	2418.14 (594.46)	3233.57 (841.41)	5217.09 (2191.57)	< 0.001	0.296	2903.89 (1062.14)	3647.57 (1785.09)	4329.18 (2179.88)	0.002	0.226	2541.16 (633.21)	3296.87 (1081.96)	5023.45 (2290.79)	< 0.001	0.881
**Copper (mg/day)**	2.29 (0.73)	2.78 (0.94)	4.62 (2.86)	< 0.001	0.078	2.51 (0.82)	2.98 (1.39)	4.21 (2.92)	< 0.001	0.366	2.35 (0.74)	2.83 (1.04)	4.51 (2.91)	< 0.001	0.149
**Manganese (mg/day)**	2.69 (0.55)	3.30 (0.90)	4.68 (1.90)	< 0.001	0.931	2.92 (0.71)	3.42 (1.32)	4.34 (1.89)	< 0.001	0.868	2.76 (0.61)	3.33 (0.98)	4.57 (1.95)	< 0.001	0.825
**Selenium (mg/day)**	0.03 (0.01)	0.05 (0.04)	0.08 (0.06)	< 0.001	0.929	0.03 (0.01)	0.05 (0.02)	0.08 (0.07)	< 0.001	0.172	0.0.03 (0.01)	0.05 (0.02)	0.08 (0.07)	< 0.001	0.738
**Fluorine (mg/day)**	7350.58 (6449.84)	10402.47 (9419.36)	8917.95 (8091.78)	0.241	0.057	8348.76 (6825.60)	8540.42 (8620.91)	9806.17 (8857.45)	0.682	0.390	8226.08 (6979.17)	9491.05 (9490.82)	9174.36 (7864.30)	0.772	0.438
**Chromium (mg/day)**	0.06 (0.03)	0.06 (0.04)	0.08 (0.05)	0.039	0.629	0.06 (0.03)	0.06 (0.04)	0.08 0.05	0.009	0.120	0.06 (0.03)	0.06 (0.04)	0.08 (0.05)	0.029	0.466
**Vitamin C (mg/day)**	106.87 (46.85)	144.64 (76.33)	211.48 (123.92)	< 0.001	0.811	127.85 (59.49)	162.74 (115.56)	172.91 (105.29)	0.095	0.137	115.46 (46.56)	140.23 (73.41)	207.82 (130.34)	< 0.001	0.431
**VitaminB1 (mg/day)**	1.30 (0.37)	1.82 (0.60)	2.7859 (1.11668)	< 0.001	0.799	1.30 (0.46)	1.75 (0.44)	2.85 (1.09)	< 0.001	**< 0.001**^a,b^	1.26 (0.35)	1.79 (0.56)	2.86 (1.05)	< 0.001	0.178
**VitaminB2 (mg/day)**	1.08 (0.32)	1.60 (0.46)	2.98 (1.66)	< 0.001	0.267	1.35 (0.65)	1.79 (0.83)	2.52 (1.79)	< 0.001	0.596	1.12 (0.39)	1.67 (0.58)	2.86 (1.71)	< 0.001	0.875
**VitaminB3 (mg/day)**	15.01 (3.47)	21.90 (5.99)	37.16 (16.17)	< 0.001	0.639	15.37 (4.84)	22.19 (6.46)	36.53 (16.39)	< 0.001	**< 0.001**^a, b^	14.61 (3.28)	21.53 (5.20)	37.94 (15.54)	< 0.001	**0.044**^b^
**VitaminB6 (mg/day)**	0.94 (0.29)	1.28 (0.40)	2.22 (1.18)	< 0.001	0.846	1.09 (0.46)	1.50 (0.87)	1.85 (1.13)	0.001	0.208	0.96 (0.29)	1.31 (0.43)	2.17 (1.21)	< 0.001	0.628
**VitaminB9 (µg/day)**	192.46 (50.72)	257.53 (69.75)	426.89	< 0.001	0.841	218.78 (76.56)	299.82 (146.93)	358.92 (208.72)	< 0.001	0.364	198.56 (53.05)	263.52 (79.62)	413.87 (217.23)	< 0.001	0.727
**VitaminB12 (µg/day)**	3.35 (1.53)	5.18 (2.45)	15.32 (24.45)	< 0.001	0.094	4.33 (2.58)	5.80 (3.20)	13.95 (24.74).	0.009	0.379	3.28 (1.34)	5.73 (2.89)	14.80 (24.62)	0.001	0.098
**VitaminB5 (mg/day)**	3.91 (1.00)	5.36 (1.27)	9.78 (5.09)	< 0.001	0.596	4.80 (2.16)	6.20 (3.51)	8.08 (5.01)	0.001	0.127	4.02 (1.10)	5.65 (1.83)	9.3 (5.30)	< 0.001	0.475
**VitaminB8 (mg/day)**	14.28 (4.93)	18.84 (7.35)	27.28 (11.41)	< 0.001	**0.043**^a^	16.81 (7.65)	20.81 (8.19)	22.84 (12.37)	0.019	0.371	15.06 (6.12)	19.67 (7.74)	25.48 (12.02)	< 0.001	0.731
**Vitamin A (RAE/day)**	806.61 (405.91)	1080.66 (678.02)	2418.41 (2234.99)	< 0.001	0.444	979.98 (579.36)	1219.61 (827.64).	2110.32 (2322.94)	0.002	0.321	833.60 (407.69)	1096.98 (642.50)	2368.61 (2279.56)	< 0.001	0.278
**Vitamin D (µg/day)**	1.31 (0.94)	1.93 (1.51)	3.21 (3.20)	< 0.001	**0.037**^a^	1.79 (1.63)	2.16 (1.88)	2.51 (2.98)	0.356	0.881	1.50 (1.21)	1.98 (1.77)	2.96 (3.11)	0.012	0.535
**Vitamin K (µg/day)**	168.54 (78.52)	232.93 (122.07)	305.61 (187.45)	< 0.001	0.167	188.90 (91.08)	221.52 (114.02)	297.16 (195.50)	0.003	0.205	181.70 (90.94)	221.66 (123.84)	306.90 (183.90)	< 0.001	0.352
**Vitamin E (mg/day)**	2.24 (0.57)	2.92 (0.98)	4.27 (1.41)	< 0.001	0.567	2.43 (0.87)	3.15 (1.08)	3.85 (1.58)	< 0.001	0.734	2.30 (0.68)	2.96 (0.97)	4.14 (1.51)	< 0.001	0.900

*P Significant at *P* < 0.05; 95th confidence intervals of the difference in parentheses. **P values are obtained from ANCOVA model after adjustment for the confounding effects of age, sex, BMI and physical activity, calorie intake

^a^post hoc Tukey signature difference between 1st tertile and 3rd tertile

^b^post hoc Tukey signature difference between 2nd tertile and 3rd tertile

**Table 4:** Biochemical parameters of study participants across different tertiels of dietary choline, betaine and total choline and betaine intake

Variables	Total choline					Total betaine					Total choline and betaine				
	T1 (*n* = 40)	T2 (*n* = 40)	T3 (*n* = 40)	*P	**P	T1 (*n* = 40)	T2 (*n* = 40)	T3 (*n* = 40)	*P	**P	T1 (*n* = 40)	T2 (*n* = 40)	T3 (*n* = 40)	*P	**P
**FBS (mg/dl)**	70.83 (8.33)	70.97 (7.80)	69.70 (6.86)	0.832	0.538	70.59 (8.00)	70.65 (7.94)	70.26 (7.15)	0.969	0.499	70.08 (8.20)	72.16 (7.23)	69.25 (7.36)	0.209	0.075
**Insulin (µ IU/ml)**	10.45 (10.09)	8.94 (5.01)	9.41 (5.97)	0.881	0.572	11.54 (10.37)	8.86 (4.17)	8.43 (5.08)	0.116	0.175	10.47 (10.13)	9.33 (5.94)	9.00 (4.97)	0.641	0.620
**TC (mg/dl)**	208.06 (61.71)	205.19 (56.79)	185.95 (53.51)	0.172	0.671	209.40 (44.93)	218.17 (65.20)	171.67 (51.25)	**< 0.001**	**0.032**^b^	206.44 (52.65)	208.44 (63.26)	184.29 (54.97)	0.110	0.150
**HDL (mg/dl)**	47.76 (11.36)	50.73 (11.88)	48.40 (11.07)	0.469	0.524	45.43 (9.34)	52.16 (11.89)	49.25 (12.05)	**0.028**	**0.049**^a^	46.48 (11.39)	50.93 (10.91)	49.45 (11.78)	0.205	0.545
**LDL (mg/dl)**	135.21 (67.72)	127.34 (59.52)	113.67 (53.70)	0.272	0.784	141.30 (46.56)	138.84 (70.47)	96.23 (52.44)	0.001	0.183	136.82 (60.70)	130.66 (61.88)	10,878 (57.24)	0.090	0.428
**TG (mg/dl)**	125.44 (80.83)	135.54 (64.16)	119.39 (63.97)	0.399	0.272	113.35 (66.02)	135.79 (75.95)	130.94 (66.45)	0.317	0.312	115.65 (81.38)	134.19 (57.36)	130.29 (69.11)	0.456	0.325
**CK (U/L)**	209.51 (67.49)	201.80 (76.43)	345.40 (543.41)	0.174	0.092	198.03 (77.09)	270.60 (333.84)	287.80 (443.93)	0.423	0.582	200.28 (69.20)	219.00 (78.44)	337.20 (544.84)	0.118	0.142
**HOMA-IR**	1.86 (1.89)	1.57 (0.91)	1.64 (1.07)	0.912	0.573	2.05 (2.00)	1.56 (0.80)	1.46 (0.87)	0.111	0.143	1.85 (1.90)	1.63 (1.07)	1.55 (0.88)	0.591	0.544
**QUICKI**	0.36 (0.03)	0.36 (0.03)	0.37 (0.04)	0.912	0.624	0.36 (0.03)	0.36 (0.03)	0.37 (0.04)	0.179	0.226	0.36 (0.03)	0.36 (0.03)	0.37 (0.04)	0.623	0.425

*FBS *Fasting blood sugar, *TC *Total cholesterol, *HDL *High-density lipoprotein, *LDL *Low-density lipoprotein, *TG *Triglycerides, *CK *Creatine kinase. *P Significant at *P* < 0.05; 95th confidence intervals of the difference in parentheses. **P values are obtained from ANCOVA model after adjustment for the confounding effects of age, sex, BMI and physical activity, calorie intake and further adjusted for muscle mass for CK level

^a^post hoc Tukey signature difference between 1st tertile and 2nd tertile

^b^post hoc Tukey signature difference between 2nd tertile and 3rd tertile

## Discussion

As far as we know, there are no other studies that have examined the interactive relationship between dietary choline and betaine and physical activity on circulating creatine kinase (CK), metabolic and glycemic markers, and anthropometric characteristics in physically active young people.

An increase in dietary betaine is associated with a decrease in TC and an increase in HDL and an increase in height, weight, MAC, WHR, FFM, MM, BM in anthropometric measurements (*P* < 0.05). In addition, there was a significant direct relationship between the total amount of dietary choline and betaine and weight, WC, WHR, FFM, and BM (*P* < 0.05). The interaction effect of dietary choline and moderate or high physical activity improved insulin resistance (*P* < 0.05). Higher dietary betaine along with high physical activity increased HDL in the highest tertile of dietary choline content compared with the lowest tertile (*P* < 0.05).

An important strength of the current study is the systematic control of all confounding factors and the accurate measurement of all anthropometric and biochemical characteristics. In population-based studies, in order to obtain more reliable results, it is essential to recognize and control the most important confounding factors. The factors that influence anthropometric and biochemical markers as confounding factors include age, sex, body mass index, physical activity, and energy intake [47, 48]. In the present study, all these confounders were adjusted for in all analyses.

Similar to our findings, in the study by Azad Bakht et al. dietary betaine was significantly related to MAC (*r* = 0.093, *P* = 0.009) and obesity risk (*P* < 0.05), but there was no association between choline and anthropometric measurements [49]. in general population. Similar to our study, Gao X et al. showed that increasing dietary betaine and dietary total choline and betaine increases FFM [35].Unlike the present study, increased WHR by increased dietary choline were observed in the study by Dibaba D et al. [24]. like the present study a study by Xiang Gao et al. showed that serum choline was positively associated with weight, BMI, and WC (r ranged from 0,09 to 0.10, and *p* < 0.05 for all) [50]. In two different studies conducted in obese adults and men, supplementation with betaine had no significant effect on the body composition of the participants [27, 51]. Increasing dietary choline and betaine significantly correlated with a reduction in BMI, waist circumference, and weight loss [52].

The results of the various studies appear to be inconsistent because of differences in the general and demographic characteristics of their populations. Some studies also differ in terms of the mode of investigation; some have used dietary choline and betaine, others have used choline or betaine supplements, and still others have examined serum levels of these micronutrients.

Insulin resistance is a multifaceted pathophysiologic condition whose development and progression are influenced by many factors. Recognizing, assessing, and monitoring these characteristics in analyzes is critical to uncovering meaningful associations. Age, gender and total daily caloric intake are among the factors that influence insulin sensitivity and should be monitored [53–56]. The interaction effect of dietary choline and moderate or high physical activity improved insulin resistance (decreasing insulin and HOMAIR and increasing QUICKI) (*P* < 0.05). In addition, increased dietary choline and betaine along with high physical activity decreased FBS in the third tertile of dietary choline and betaine compared with the first tertile (*P* < 0.05). The results of the study by X. Gao et al. showed an inverse correlation between dietary choline and betaine and fasting glucose and insulin levels and HOMA-IR (*r* = -0.08 to -0.27 for choline and *r* = -0.06 to -0.16 for betaine; *P* < 0.05), indicating that higher intake was associated with lower insulin resistance. Choline and betaine were positively related to QUICKI (*r* = 0.16–0.25 for choline and *r* = 0.11–0.16 for betaine; *P* < 0.01), indicating higher insulin sensitivity [6]. Choline supplement could alleviate inflammation and suppress oxidative stress, which play important roles in the development of IR, they noted, before adding that choline can also be metabolized to betaine, which could impact IR via a couple of different routes, including improving signaling pathways for glycogen synthesis and enhancing insulin sensitivity in fat tissue. Betaine may also reduce inflammatory markers levels [57–59].

In the present investigation, Increasing dietary betaine decreased serum levels TC and increased HDL (*P* < 0.05). Higher dietary betaine along with high physical activity increased HDL in the highest tertile of dietary choline compared with the lowest tertile (*P* < 0.05). In other research, there was an adverse association between serum betaine and some lipid profile factors (triglycerides, non-HDL cholesterol, and HDL cholesterol) [60]. Participants who were involved in a lipid clinic showed that betaine had an adverse relationship with apolipoprotein B (Apo B) and body fat [61, 62]. Current research demonstrates the function of BHMT (betaine homocysteine S-methyltransferase) in lipid metabolism, such that dietary induction of BHMT in mice causes elevated liver VLDL secretion, ApoB, ApoB mRNA, and triglyceride production [63]. Betaine homocysteine S-methyltransferase, as the most abundant protein in the liver of mammals, in addition to homocysteine remethylation, binds to membranes and is related to various proteins. Therefore, it can be concluded that BHMT probably indicates a metabolic link between 1-carbon metabolism and lipids [60, 64].

Creatine phosphokinase is an enzyme that is found in skeletal and cardiac muscle cells (MM and MB isoform) and brain tissue (BB isoform). Higher CK in the blood is typical because of muscle damage or muscular dystrophy [65–67]. Adequate dietary choline is important for maintaining muscle cell integrity, as a choline-deficient diet is associated with increased serum creatine phosphokinase in humans [17]. Progressive muscular dystrophy in rabbits is associated with choline deficiency [65, 68–70]. Deficiency of choline through the reduction of phosphatidylcholine in muscle cells induces apoptosis, increases the fragility of the membrane, and eventually leaks creatine phosphokinase to the outside of the cell. Additionally, receiving a low choline diet in men significantly increased the activity of serum creatine phosphokinase [17]. In various studies, it has been shown that there is a relationship between serum creatine kinase levels and some metabolic and glycemic indicators. In other words, elevated serum creatine kinase is associated with obesity, increased WC and BMI, WC, WHR and increased insulin resistance, and increased risk of heart disease [71–73]. However, serum creatine kinase levels depend on various factors such as age, sex, muscle mass, and physical activity [74–76]. In the present study, the group with the highest tertile of dietary choline and betaine had higher serum creatine kinase levels, although this association was not significant. This relationship can be explained by the fact that the group that received the most choline and betaine had the highest physical activity and muscle mass.

This is a cross-sectional study that cannot establish a cause-and-effect relationship.There is a need for another longitudinal study to fill this knowledge gap. In addition, although several factors were fully controlled in our analysis, including age, sex, BMI, physical activity, and caloric intake, genetic factors and unknown or poorly measured factors were not fully eliminated. The cross-sectional design of the current study makes it difficult to draw causal conclusions; In addition, a semiquantitative dietary assessment questionnaire was used that could introduce recall bias because of its subjective nature; however, the validity and reliability of the questionnaire have been confirmed in previous studies. Other strengths of this study include the numerous variables examined.

## Conclusions

In our cross-sectional study of Iranian youth, the interaction effect of dietary choline and moderate or high physical activity improved insulin resistance (*P* < 0.05). Higher dietary betaine along with high physical activity increased HDL in the highest tertile of dietary choline compared with the lowest tertile (*P* < 0.05). Additionally, increased dietary choline and betaine together with high physical activity decreased FBS in the third tertile of dietary choline and betaine compared with the first tertile (*P* < 0.05).

## Supplementary Information

**Additional file 1.**

## Data Availability

The datasets used and/or analyzed during the current study available from the corresponding author on reasonable request.

## Abbreviations

FFQ: Food frequency questionnaire
PA: Physical activity
BMI: Body mass index
ANOVA: Analysis of variance
ANCOVA: Analysis of covariance
BIA: Bioelectric impedance analysis
MAC: Mid arm circumference
WC: Waist circumference
WHR: Waist to hip ratio
FFA: Free fatty acids
CK: Creatin kinase
TC: Total cholesterol
LDL: Low density lipoprotein
HDL: High density lipoprotein
TG: Trygliserid

## References

[1] World Health O (2006). Global strategy on Diet, Physical Activity and Health: a framework to monitor and evaluate implementation.

[2] Milan Z, Titta K, Razvan Constantin D, Nikola A, Bojan B, Dan Iulian A (2022). Leisure-time physical activity and all-cause mortality: a systematic review. Revista de Psicología del Deporte. (Journal of Sport Psychology).

[3] Aparicio-Ting FE, Farris M, Courneya KS, Schiller A, Friedenreich CM (2015). Predictors of physical activity at 12 month follow-up after a supervised exercise intervention in postmenopausal women. Int J Behav Nutr Phys Activity.

[4] Miles L (2007). Physical activity and health. Nutr Bull.

[5] Leman MA, Claramita M, Rahayu GR (2021). Predicting factors on modeling health behavior: a systematic review. Am J Health Behav.

[6] Gao X, Wang Y, Sun G (2017). High dietary choline and betaine intake is associated with low insulin resistance in the Newfoundland population. Nutrition.

[7] Jafari S, Nabavi SM, Silva AS (2019). Chapter 2.4 - choline. Nonvitamin and nonmineral.

[8] PAVLOS S (2022). Medicinal plants against obesity: a Met-Analysis of Literature. J Complement Med Res.

[9] Ueland PM (2011). Choline and betaine in health and disease. J Inherit Metab Dis.

[10] Vennemann FB, Ioannidou S, Valsta LM, Dumas C, Ocké MC, Mensink GB (2015). Dietary intake and food sources of choline in european populations. Br J Nutr.

[11] Virtanen JK, Tuomainen T-P, Voutilainen S (2020). Dietary intake of choline and phosphatidylcholine and risk of type 2 diabetes in men: the Kuopio Ischaemic Heart Disease risk factor study. Eur J Nutr.

[12] Wallace TC, Fulgoni VL (2016). Assessment of Total Choline Intakes in the United States. J Am Coll Nutr.

[13] Leermakers ET, Moreira EM, Kiefte-de Jong JC, Darweesh SK, Visser T, Voortman T (2015). Effects of choline on health across the life course: a systematic review. Nutr Rev.

[14] Li P (2022). A study on the psychological and social factors related to exercise addiction among middle-aged and elderly people. Revista de Psicología del Deporte. (Journal of Sport Psychology).

[15] Craig SA (2004). Betaine in human nutrition. Am J Clin Nutr.

[16] Moretti A, Paoletta M, Liguori S, Bertone M, Toro G, Iolascon G. Choline: An Essential Nutrient for Skeletal Muscle. Nutrients. 2020;12(7):1-3. 10.3390/nu12072144 PMC7400816 32708497

[17] da Costa KA, Badea M, Fischer LM, Zeisel SH (2004). Elevated serum creatine phosphokinase in choline-deficient humans: mechanistic studies in C2C12 mouse myoblasts. Am J Clin Nutr.

[18] Mougios V (2007). Reference intervals for serum creatine kinase in athletes. Br J Sports Med.

[19] Hyder KM, Mohan J, Varma V, Sivasankaran P, Raja D (2021). Effects of muscle–specific exercises compared to existing interventions on insulin resistance among Prediabetes Population of South India. J Nat Sci Biology Med.

[20] Crandall JP, Knowler WC, Kahn SE, Marrero D, Florez JC, Bray GA (2008). The prevention of type 2 diabetes. Nat Clin Pract Endocrinol Metab.

[21] Hu FB (2011). Globalization of diabetes: the role of diet, lifestyle, and genes. Diabetes Care.

[22] Schroeder K, Kubik MY, Sirard JR, Lee J, Fulkerson JA (2020). Sleep is inversely associated with sedentary time among youth with obesity. Am J Health Behav.

[23] Paul R, Mukkadan J (2020). Modulation of blood glucose, oxidative stress, and anxiety level by controlled vestibular stimulation in prediabetes. J Nat Sci Biol Med.

[24] Dibaba DT, Johnson KC, Kucharska-Newton AM, Meyer K, Zeisel SH, Bidulescu A (2020). The association of dietary choline and betaine with the risk of type 2 diabetes: the atherosclerosis risk in Communities (ARIC) study. Diabetes Care.

[25] Wu G, Zhang L, Li T, Lopaschuk G, Vance DE, Jacobs RL (2012). Choline Deficiency attenuates Body Weight Gain and improves glucose tolerance in ob/ob mice. J Obes.

[26] Zhou L, Li X, Li S, Wen X, Peng Y, Zhao L (2021). Relationship between dietary choline intake and diabetes mellitus in the National Health and Nutrition Examination Survey 2007-2010. J Diabetes.

[27] Schwab U, Törrönen A, Toppinen L, Alfthan G, Saarinen M, Aro A (2002). Betaine supplementation decreases plasma homocysteine concentrations but does not affect body weight, body composition, or resting energy expenditure in human subjects. Am J Clin Nutr.

[28] Ross AC, Caballero B, Cousins R. Modern nutrition in health and disease. 11th ed. Jones & Bartlett Learning; 2020. p. 416–23.

[29] Parker N, Atrooshi D, Lévesque L, Jauregui E, Barquera S, Taylor, JLy (2016). Physical activity and anthropometric characteristics among Urban Youth in Mexico: a cross-sectional study. J Phys Activity Health.

[30] Guerra ZC, Moore JR, Londoño T, Castro Y (2022). Associations of acculturation and gender with obesity and physical activity among Latinos. Am J Health Behav.

[31] Rostamizadeh M, Elmieh A, Rahmani Nia F (2019). Effects of aerobic and resistance exercises on anthropometric indices and osteocalcin, leptin, adiponectin levels in overweight men. J Arak Univ Med Sci.

[32] Młodzik-Czyżewska M, Malinowska A, Chmurzyńska A (2020). Associations between choline intake, body composition, lipid profile, and liver status in healthy adults. Proc Nutr Soc.

[33] Cholewa JM, Wyszczelska-Rokiel M, Glowacki R, Jakubowski H, Matthews T, Wood R (2013). Effects of betaine on body composition, performance, and homocysteine thiolactone. J Int Soc Sports Nutr.

[34] Cholewa JM, Hudson A, Cicholski T, Cervenka A, Barreno K, Broom K (2018). The effects of chronic betaine supplementation on body composition and performance in collegiate females: a double-blind, randomized, placebo controlled trial. J Int Soc Sports Nutr.

[35] Gao X, Wang Y, Randell E, Pedram P, Yi Y, Gulliver W (2016). Higher dietary choline and betaine intakes are associated with better body composition in the adult population of Newfoundland, Canada. PLoS ONE.

[36] Nikniaz L, Tabrizi J, Sadeghi-Bazargani H, Farahbakhsh M, Tahmasebi S, Noroozi S. Reliability and relative validity of short-food frequency questionnaire. Br Food J. 2017;119(6):1337-48.

[37] Kristine Y, Patterson SAB, Juhi R, Williams JC. Howe aJMH. USDA Database for the Choline Content of Common Foods; January 2008.

[38] Zeisel SH, Mar MH, Howe JC, Holden JM (2003). Concentrations of choline-containing compounds and betaine in common foods. J Nutr.

[39] Cho E, Holmes MD, Hankinson SE, Willett WC (2010). Choline and betaine intake and risk of breast cancer among post-menopausal women. Br J Cancer.

[40] Azadbakht L, Esmaillzadeh A, Macro, Intake M-N (2012). Food Groups Consumption and Dietary Habits among female students in Isfahan University of Medical Sciences. Iran Red Crescent Med J.

[41] U.S. Department of Agriculture Food data. [Internet]. 2023 [cited 21 March 2021]. Available from: https://fdc.nal.usda.gov.

[42] Pehrsson PR, Patterson KY, Spungen JH, Wirtz MS, Andrews KW, Dwyer JT (2016). Iodine in food- and dietary supplement-composition databases. Am J Clin Nutr.

[43] Fukuyama N, Homma K, Wakana N, Kudo K, Suyama A, Ohazama H (2007). Validation of the Friedewald equation for evaluation of plasma LDL-cholesterol. J Clin Biochem Nutr.

[44] Matthews DR, Hosker JP, Rudenski AS, Naylor BA, Treacher DF, Turner RC (1985). Homeostasis model assessment: insulin resistance and beta-cell function from fasting plasma glucose and insulin concentrations in man. Diabetologia.

[45] Katz A, Nambi SS, Mather K, Baron AD, Follmann DA, Sullivan G (2000). Quantitative insulin sensitivity check index: a simple, accurate method for assessing insulin sensitivity in humans. J Clin Endocrinol Metab.

[46] Gokalp G, Berksoy E, Bardak S, Demir G, Demir S, Anil M (2021). Is there a relationship between thyroid hormone levels and suicide attempt in adolescents?. Archives of Clinical Psychiatry (São Paulo).

[47] Jackson AS, Stanforth PR, Gagnon J, Rankinen T, Leon AS, Rao D (2002). The effect of sex, age and race on estimating percentage body fat from body mass index: the Heritage Family Study. Int J Obes.

[48] Bergens O, Veen J, Montiel-Rojas D, Edholm P, Kadi F, Nilsson A (2020). Impact of healthy diet and physical activity on metabolic health in men and women: Study Protocol Clinical Trial (SPIRIT compliant). Medicine.

[49] Jafari A, Jalilpiran Y, Suitor K, Bellissimo N, Azadbakht L (2021). The association of dietary choline and betaine and anthropometric measurements among iranian children: a cross-sectional study. BMC Pediatr.

[50] Gao X, Randell E, Zhou H, Sun G (2018). Higher serum choline and betaine levels are associated with better body composition in male but not female population. PLoS ONE.

[51] del Favero S, Roschel H, Artioli G, Ugrinowitsch C, Tricoli V, Costa A (2012). Creatine but not betaine supplementation increases muscle phosphorylcreatine content and strength performance. Amino Acids.

[52] Díez-Ricote L, San-Cristobal R, Concejo MJ, Martínez-González M, Corella D, Salas-Salvadó J et al. One-year longitudinal association between changes in dietary choline or betaine intake association with cardiometabolic variables in the PREDIMED-Plus trial. Am J Clin Nutr. 2022. 10.1093/ajcn/nqac255 PMC9761742 36124652

[53] Geer EB, Shen W (2009). Gender differences in insulin resistance, body composition, and energy balance. Gend Med.

[54] Martins VF, Tahvilian S, Kang JH, Svensson K, Hetrick B, Chick WS (2018). Calorie Restriction-Induced increase in skeletal muscle insulin sensitivity is not prevented by overexpression of the p55α subunit of phosphoinositide 3-Kinase. Front Physiol.

[55] Chen Z, Watanabe RM, Stram DO, Buchanan TA, Xiang AH (2014). High calorie intake is associated with worsening insulin resistance and β-cell function in hispanic women after gestational diabetes mellitus. Diabetes Care.

[56] Sowmya S, Rao RS, Prasad K. Prediction of metastasis in oral squamous cell carcinoma through phenotypic evaluation and gene expression of E-cadherin, β-catenin, matrix metalloproteinase-2, and matrix metalloproteinase-9 biomarkers with clinical correlation. J Carcinog. 2020;19. 10.4103/jcar.JCar_8_20 PMC7511891 33033464

[57] Detopoulou P, Panagiotakos DB, Chrysohoou C, Fragopoulou E, Nomikos T, Antonopoulou S (2010). Dietary antioxidant capacity and concentration of adiponectin in apparently healthy adults: the ATTICA study. Eur J Clin Nutr.

[58] Mehta AK, Singh BP, Arora N, Gaur SN (2010). Choline attenuates immune inflammation and suppresses oxidative stress in patients with asthma. Immunobiology.

[59] Xavier J, Farias CP, Soares MSP, Silveira GdO, Spanevello RM, Yonamine M (2021). Ayahuasca prevents oxidative stress in a rat model of depression elicited by unpredictable chronic mild stress. Archives of Clinical Psychiatry (São Paulo).

[60] Konstantinova SV, Tell GS, Vollset SE, Nygård O, Bleie Ø, Ueland PM (2008). Divergent associations of plasma choline and betaine with components of metabolic syndrome in middle age and elderly men and women. J Nutr.

[61] Lever M, George PM, Dellow WJ, Scott RS, Chambers ST (2005). Homocysteine, glycine betaine, and N,N-dimethylglycine in patients attending a lipid clinic. Metabolism.

[62] Khan W, Augustine D, Rao RS, Patil S, Awan KH, Sowmya SV et al. Lipid metabolism in cancer: a systematic review. J Carcinog. 2021;20. 10.4103/jcar.JCar_15_20 PMC8312377 34321955

[63] Sparks JD, Collins HL, Chirieac DV, Cianci J, Jokinen J, Sowden MP (2006). Hepatic very-low-density lipoprotein and apolipoprotein B production are increased following in vivo induction of betaine-homocysteine S-methyltransferase. Biochem J.

[64] Pajares MA, Pérez-Sala D (2006). Betaine homocysteine S-methyltransferase: just a regulator of homocysteine metabolism?. Cell Mol Life Sci.

[65] Hove EL, Copeland DH (1954). Progressive muscular dystrophy in rabbits as a result of chronic choline deficiency. J Nutr.

[66] Rendt K (2001). Inflammatory myopathies: narrowing the differential diagnosis. Cleve Clin J Med.

[67] Arahata K (2000). Muscular dystrophy. Neuropathology.

[68] Hove EL, Copeland DH, Salmon WD (1954). Choline deficiency in the rabbit. J Nutr.

[69] Pellet H, Pellet M, Brun M, Minaire E (1967). [Biological study of an experimental myopathy of dietary origin in rabbits]. Pathol Biol.

[70] Pellet H (1965). Brun. [Experimental myopathy induced by choline deficiency]. Lyon Med.

[71] Kriketos AD, Pan DA, Lillioja S, Cooney GJ, Baur LA, Milner MR (1996). Interrelationships between muscle morphology, insulin action, and adiposity. Am J Physiol.

[72] Simoneau JA, Colberg SR, Thaete FL, Kelley DE (1995). Skeletal muscle glycolytic and oxidative enzyme capacities are determinants of insulin sensitivity and muscle composition in obese women. Faseb j.

[73] Bouchard C (1995). Skeletal muscle metabolism and body Fat Content in Men and Women. Obes Res.

[74] Kim EJ, Wierzbicki AS (2021). Investigating raised creatine kinase. BMJ.

[75] Sharif YH. Serum leptin level-insulin resistance-based correlation in polycystic ovary syndrome obese and non-obese sufferer female. J Popul Ther Clin Pharmacol. 2022;29(2):e11-e9. 10.47750/jptcp.2022.916 35848192

[76] Patterson YK, et al. USDA Database for the Choline Content of Common Foods. U.S. Department of Agriculture; 2004. 56d100fa-c289-4fc7-a77b-a2cce73e99d9.

