# Supplementary material for: The interactive relationship of dietary choline and betaine with physical activity on circulating creatine kinase (CK), metabolic and glycemic markers, and anthropometric characteristics in physically active young individuals
Source: BMC Endocr Disord. 2023 Jul 25;23:158. doi: 10.1186/s12902-023-01413-3 (PMC10367233; doi:10.1186/s12902-023-01413-3)
Supplement: Supplementary file 1 — Additional file 1. [file 12902_2023_1413_MOESM1_ESM.docx]

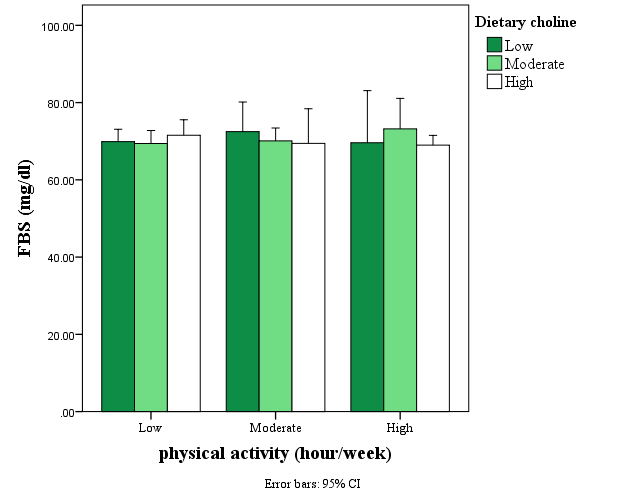

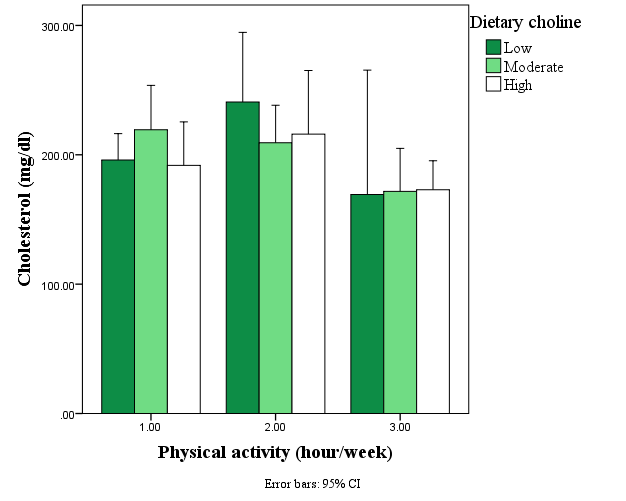


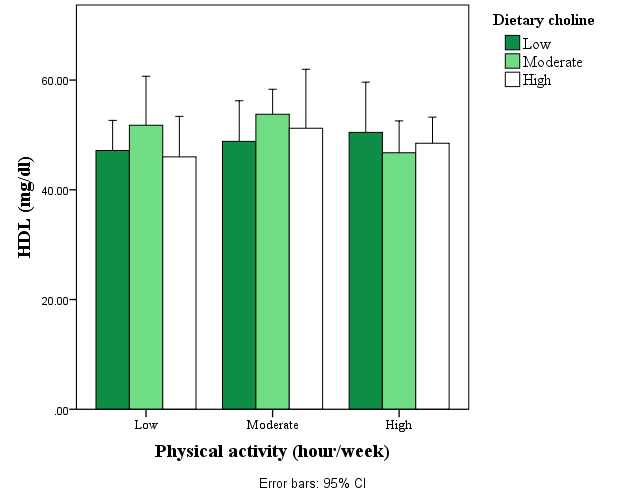

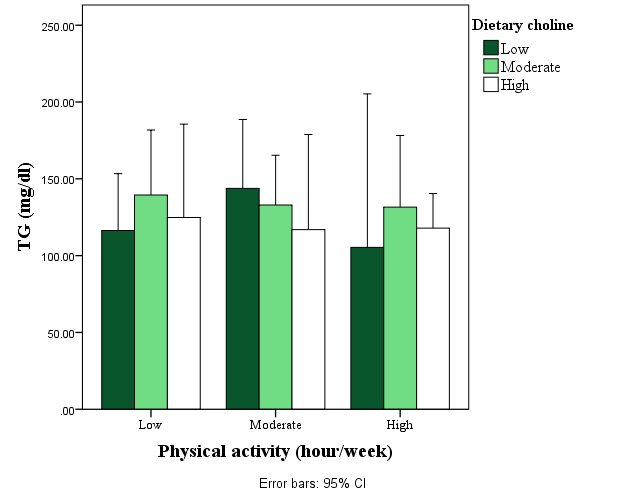


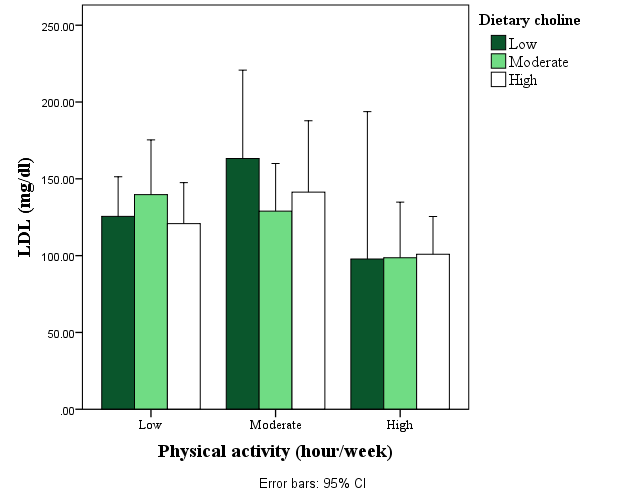

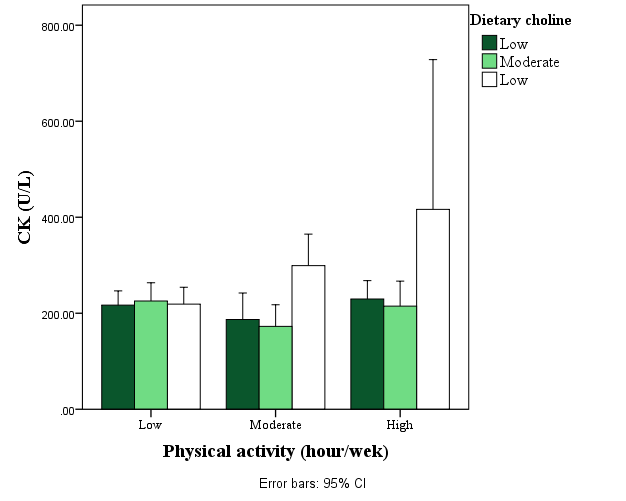


**Sup. Figure 1.** The interaction between dietary choline and physical activity on biochemical markers. FBS, fasting blood sugar; TC, total cholesterol; HDL, high-density lipoprotein; LDL, low*-*density lipoprotein; TG, triglycerides; CK, creatine kinase. P value of all Figures is non-significant (P > .05).


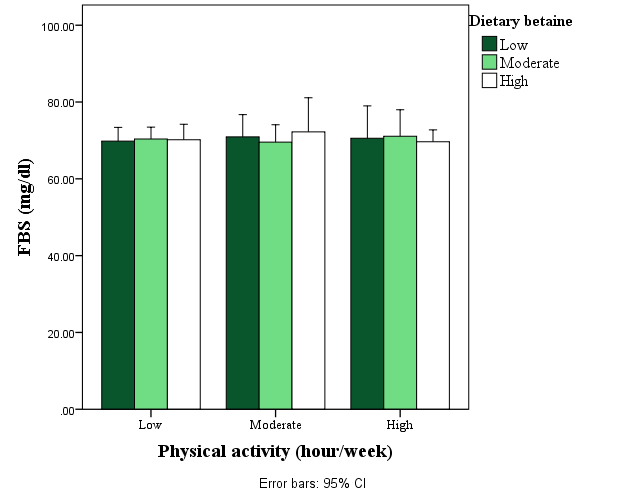

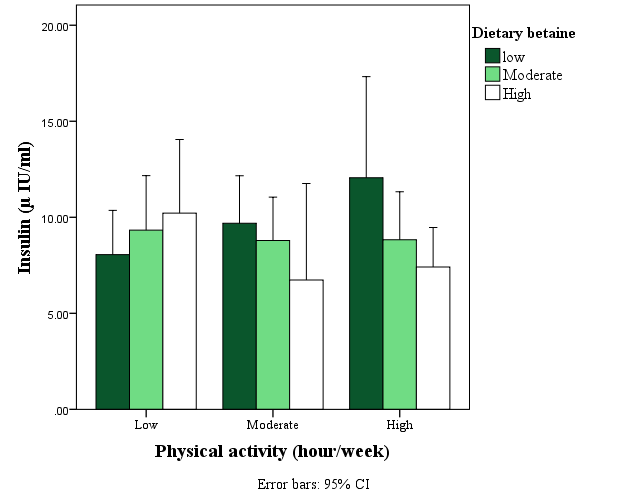


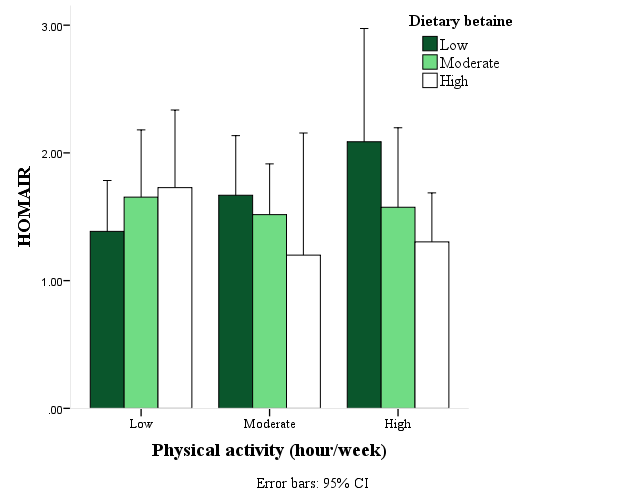

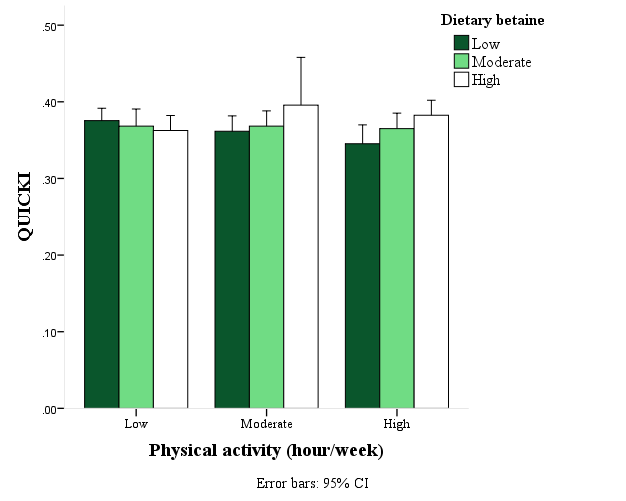


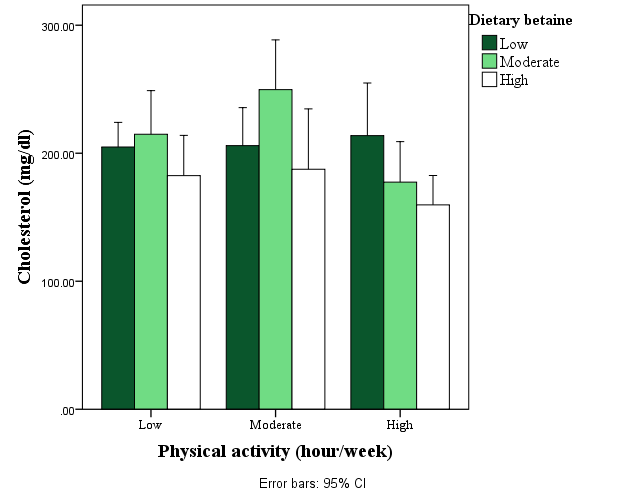

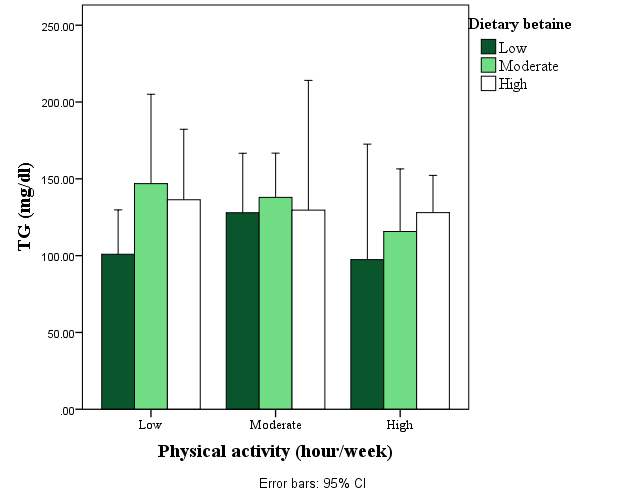


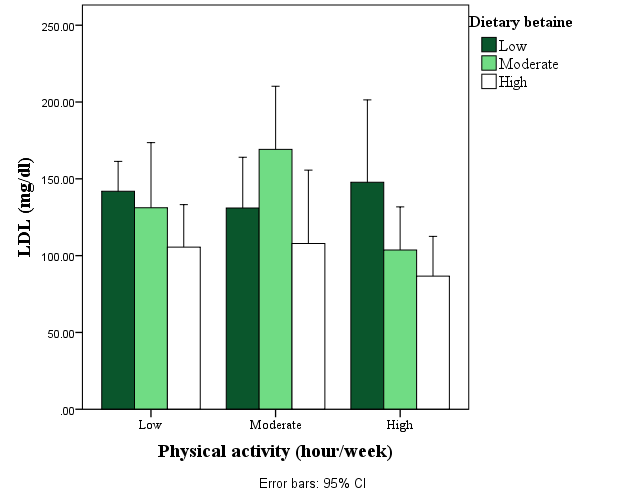

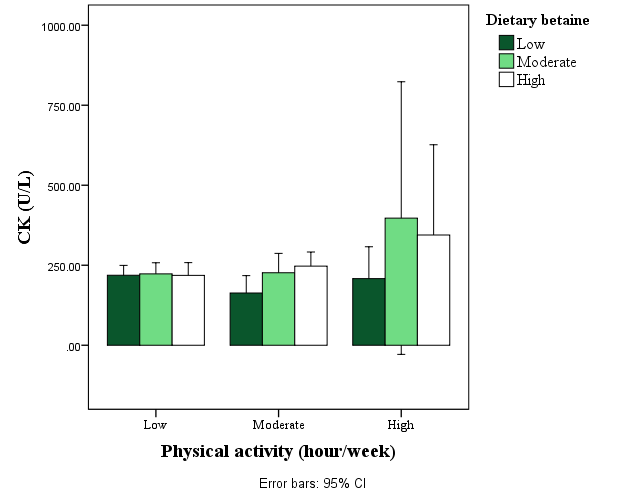


**Sup. Figure 2.** The interaction between dietary betaine and physical activity on biochemical markers. FBS, fasting blood sugar; TC, total cholesterol; LDL, low*-*density lipoprotein; TG, triglycerides; CK, creatine kinase. P value of all Figures is non-significant (P > .05).


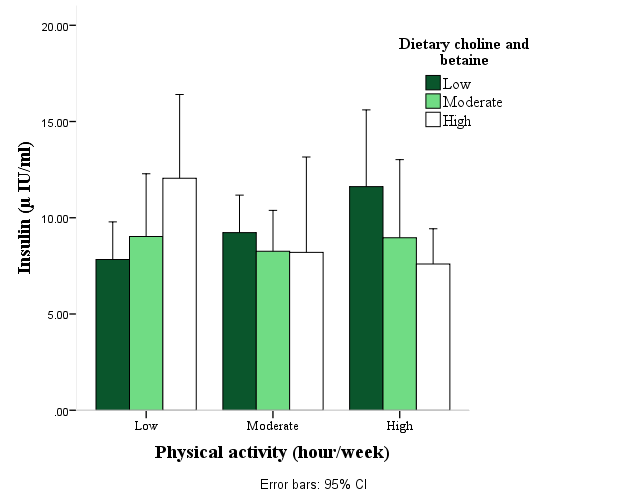

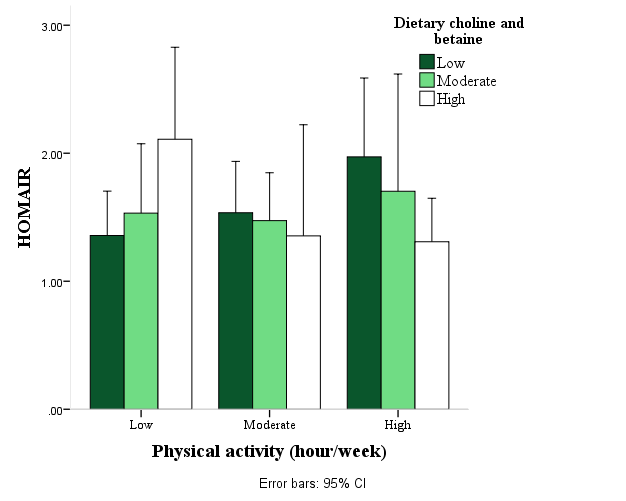


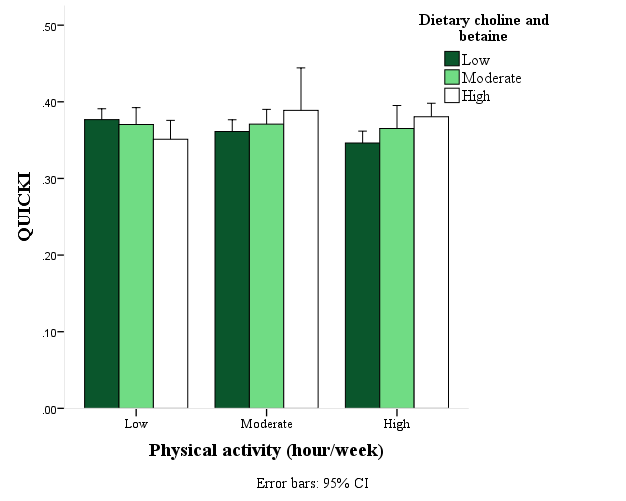

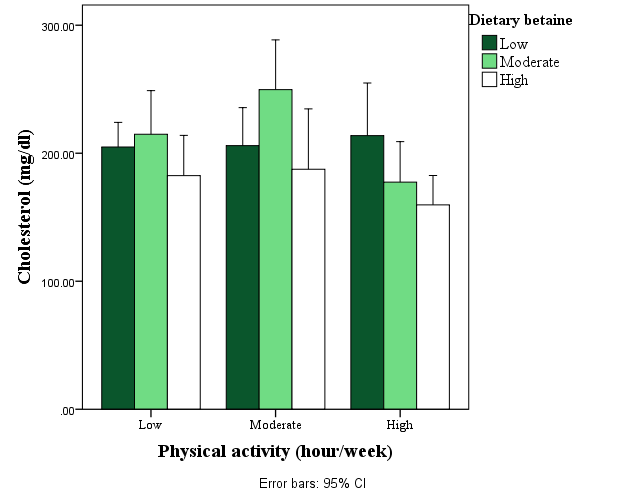


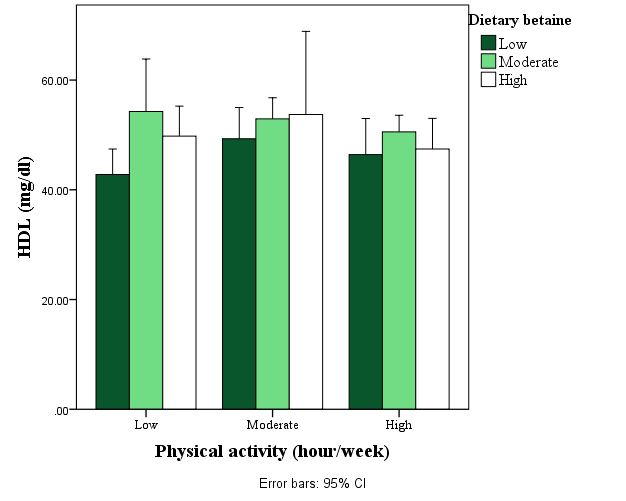

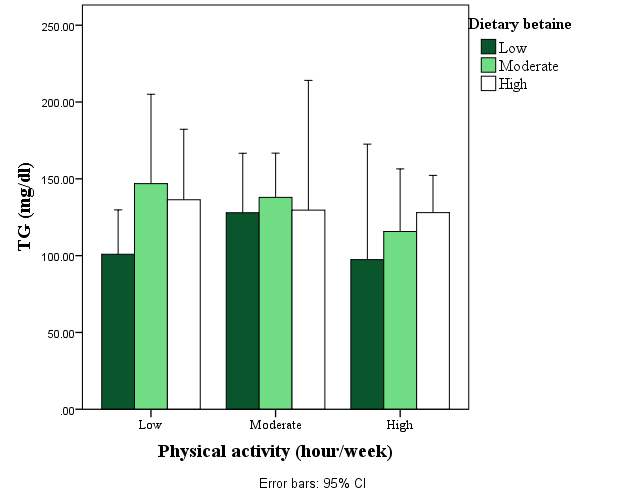


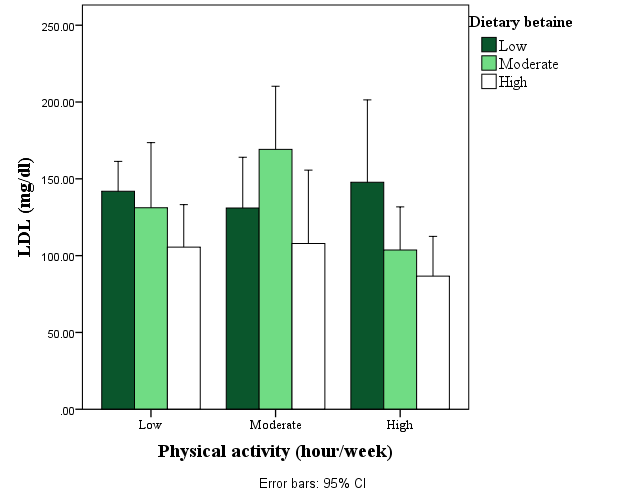

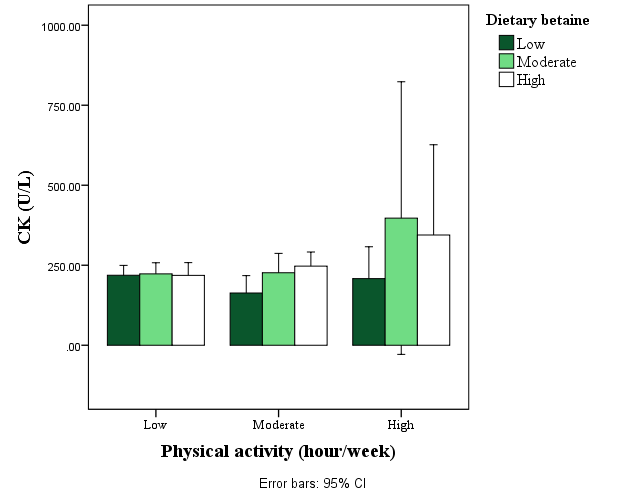


**Sup. Figure 3.** The interaction between dietary choline and betaine and physical activity on biochemical markers. TC, total cholesterol; HDL, high-density lipoprotein; LDL, low*-*density lipoprotein; TG, triglycerides; CK, creatine kinase. P value of all Figures is non-significant (P > .05).
